# Perceived stress as a predictor of eating behavior during the 3-year PREVIEW lifestyle intervention

**DOI:** 10.1038/s41387-022-00224-0

**Published:** 2022-11-05

**Authors:** Elli Jalo, Hanna Konttinen, Margriet Westerterp-Plantenga, Tanja Adam, Mathijs Drummen, Maija Huttunen-Lenz, Pia Siig Vestentoft, J. Alfredo Martinez, Svetoslav Handjiev, Ian Macdonald, Jennie Brand-Miller, Sally Poppitt, Nils Swindell, Tony Lam, Santiago Navas-Carretero, Teodora Handjieva-Darlenska, Moira Taylor, Roslyn Muirhead, Marta P. Silvestre, Anne Raben, Mikael Fogelholm

**Affiliations:** 1 Department of Food and Nutrition, University of Helsinki, Helsinki, Finland; 2 Faculty of Social Sciences, University of Helsinki, Helsinki, Finland; 3 Department of Nutrition and Movement Sciences, NUTRIM, School of Nutrition and Translational Research in Metabolism, Maastricht University, Maastricht, The Netherlands; 4 Institute of Nursing Science, University of Education Schwäbisch Gmünd, Schwäbisch, Gmünd, Germany; 5 Department of Nutrition, Exercise and Sports, Faculty of Science, University of Copenhagen, Frederiksberg, Denmark; 6 Centre for Nutrition Research, School of Pharmacy and Nutrition, University of Navarra, Pamplona, Navarra, Spain; 7 Centro de Investigación Biomédica en Red Fisiopatología de la Obesidad y Nutrición (CIBERObn), Instituto de Salud Carlos III, Madrid, Spain; 8 IdiSNA, Instituto de Investigación Sanitaria de Navarra, Pamplona, Navarra, Spain; 9 Precision Nutrition and Cardiometabolic Health Program. IMDEA-Food Institute (Madrid Institute for Advanced Studies), CEI UAM + CSIC, Madrid, Spain; 10 Department of Pharmacology and Toxicology, Medical University of Sofia, Sofia, Bulgaria; 11 Division of Physiology, Pharmacology and Neuroscience, School of Life Sciences, Queen’s Medical Centre, National Institute for Health Research (NIHR) Nottingham Biomedical Research Centre, Nottingham, UK; 12 MRC/ARUK Centre for Musculoskeletal Ageing Research, ARUK Centre for Sport, Exercise and Osteoarthritis, National Institute for Health Research (NIHR) Nottingham Biomedical Research Centre, Nottingham, UK; 13 School of Life and Environmental Sciences and Charles Perkins Centre, University of Sydney, Sydney, NSW Australia; 14 Human Nutrition Unit, School of Biological Sciences, Department of Medicine, University of Auckland, Auckland, New Zealand; 15 Applied Sports Technology, Exercise, and Medicine (A-STEM) Research Centre, College of Engineering, Swansea University, Swansea, UK; 16 NetUnion SARL, Lausanne, Switzerland; 17 Centro de Investigaçao em Tecnologias e Serciços de Saûde (CINTESIS), NOVA Medical School, NOVA University of Lisbon, Lisbon, Portugal; 18 Steno Diabetes Center Copenhagen, Gentofte, Denmark

**Keywords:** Risk factors, Lifestyle modification, Nutrition, Patient education, Weight management

## Abstract

**Background:**

To better support participants to achieve long-lasting results within interventions aiming for weight loss and maintenance, more information is needed about the maintenance of behavioral changes. Therefore, we examined whether perceived stress predicts the maintenance of changes in eating behavior (flexible and rigid restraint of eating, disinhibition, and hunger).

**Methods:**

The present study was a secondary analysis of the PREVIEW intervention including participants with overweight (BMI ≥ 25 kg/m^2^) at baseline and high risk of type 2 diabetes (*n* = 1311). Intervention included a 2-month low-energy diet phase and a 34-month subsequent weight maintenance phase. The first 6 months were considered an active behavior change stage and the remaining 2.5 years were considered a behavior maintenance stage. Eating behavior was measured using the Three Factor Eating Questionnaire and stress using the Perceived Stress Scale. The associations between stress and eating behavior were analyzed using linear mixed effects models for repeated measurements.

**Results:**

Perceived stress measured after the active behavior change stage (at 6 months) did not predict changes in eating behavior during the behavior maintenance stage. However, frequent high stress during this period was associated with greater lapse of improved flexible restraint (*p* = 0.026). The mean (SD) change in flexible restraint from 6 to 36 months was −1.1 (2.1) in participants with frequent stress and −0.7 (1.8) in participants without frequent stress (Cohen’s d_s_ (95% CI) = 0.24 (0.04–0.43)). Higher perceived stress at 6 months was associated with less flexible restraint and more disinhibition and hunger throughout the behavior maintenance stage (all *p* < 0.001).

**Conclusions:**

Perceived stress was associated with features of eating behavior that may impair successful weight loss maintenance. Future interventions should investigate, whether incorporating stress reduction techniques results in more effective treatment, particularly for participants experiencing a high stress level.

## Introduction

Lifestyle interventions are efficacious in the treatment of obesity and concomitantly in decreasing the risk of morbidities linked to obesity [1]. However, maintaining weight loss and the achieved risk reduction continues to be a challenge [2]. This most likely results from a gradual return to old lifestyle habits [3]. Deeper understanding about factors associated with maintenance of changed behavior is essential for the development of more efficient treatment.

One key target in lifestyle interventions aiming for weight loss and maintenance is eating behavior. The Three Factor Eating Questionnaire (TFEQ) measures three dimensions of eating behavior, which reflect intentional suppression of food intake to control body weight (cognitive restraint), tendency to overeat in response to certain external and internal stimuli (disinhibition), and subjective feelings of hunger and food cravings (hunger) [4]. In the context of relatively short-term (1 year or shorter) weight loss interventions, an increase in cognitive restraint, together with decreases in disinhibition and hunger have been consistently associated with a greater weight loss [5–8]. In addition, one study reported that a sustained decrease in uncontrolled eating (similar to disinhibition) was associated with better weight maintenance after an initial weight loss up to 3 years [9]. We have also previously shown in the PREVIEW study that cognitive restraint was negatively, and disinhibition and hunger were positively associated with BMI throughout the 3-year weight loss and maintenance intervention [10].

Because previous studies have been rather short-term and focused on predicting weight change with eating behavior [5–8], little is known about factors predicting long-term maintenance of changes in eating behavior. One potential factor is stress, which is increasingly common in our current society [11]. Stress is defined as a state in which environmental demands exceed the adaptive capacity of an individual leading to biological, psychological, and behavioral responses with potential health effects [12]. Focal biological response includes activation of hypothalamic-pituitary-adrenal axis resulting in elevated cortisol that is linked to increased appetite [13], which potentially makes it harder to maintain low levels of disinhibition and hunger. Additionally, maintaining behavior change may require considerable cognitive resources and self-regulation, which are potentially diminished when under stress [11].

Perceived stress describes subjective appraisal of the stressfulness of situations in one’s life [14]. In cross-sectional studies, high perceived stress has been associated with less cognitive restraint in general [15], but higher degree of rigid restraint [16]. This dimension of restraint, referring to strict all-or-nothing approach to eating is associated with higher eating disinhibition, and may thus be less beneficial than flexible restraint, which is characterized by moderate control overeating without deprivation and guilt [17]. Furthermore, perceived stress has been associated with higher tendencies of overeating, i.e. disinhibition and hunger [16, 18], as well as uncontrolled and emotional eating [15]. These cross-sectional findings indicate that perceived stress associates with features of eating behavior linked with less successful weight loss and maintenance in intervention studies [5–10, 19, 20]. However, little is known whether perceived stress associates with the maintenance of changes in eating behavior during a lifestyle intervention. The present study aims to contribute filling this gap in the current knowledge.

Our study utilizes the data from the 3-year PREVIEW intervention (PREVention of diabetes through lifestyle Intervention and population studies in Europe and around the World), which was designed to test the effectiveness of two diets and two physical activity programs for type 2 diabetes prevention and weight maintenance after an initial low-energy diet induced weight loss in participants with overweight and pre-diabetes [21]. In the present secondary observational analysis, the two main objectives were: First, to examine, whether perceived stress measured after an active behavior change stage (at 6 months) predicted the maintenance of changes in eating behavior (flexible and rigid restraint of eating, disinhibition and hunger) during a behavior maintenance stage (the remaining 2.5 years). Second, whether frequently experienced stress during the behavior maintenance stage was associated with the changes in eating behavior. To link the changes in eating behavior with the main target of the intervention (i.e., weight loss maintenance), we also examined the association between 3-year weight reduction success and changes in eating behavior during the whole intervention.

## Participants and methods

### The PREVIEW participants and design

The recruitment and design [21], and main results [22] of the PREVIEW intervention (ClinicalTrials.gov NCT01777893) have been reported in detail previously. Adult (25–70 years) men and women with overweight (BMI ≥ 25 kg/m^2^) and pre-diabetes were recruited from June 2013 to February 2015 via newspaper, radio, and television advertisements and by primary and occupational health care providers. Pre-screening was conducted via telephone and potentially eligible participants (*n* = 5472, Supplementary Fig. 1) were invited to a screening visit to confirm pre-diabetes according to the criteria of the American Diabetes Association [23]. For more details regarding inclusion/exclusion criteria, see Fogelholm et al [21]. The intervention was conducted similarly in eight countries: Denmark, Finland, The Netherlands, the UK, Spain, Bulgaria, Australia, and New Zealand. The local Human Ethics Committees reviewed the study protocol at each of the intervention centers. All participants provided written informed consent prior to any screening procedures.

The 3-year intervention consisted of two phases (Supplementary Fig. 2). Intervention started with a 2-month weight loss phase using commercial low-energy diet products (The Cambridge Weight Plan®) to achieve daily energy intake of 3.4 MJ [24]. Because the main objectives of the intervention included weight maintenance, ≥8% weight loss was required for continuation to a 34-month weight maintenance phase. Eligible participants (*n* = 1857) were randomized to follow one of two intervention diets (moderate-protein, moderate-glycemic index (GI) diet aiming at 15 E% of protein, 55 E% of carbohydrate, and GI > 56 or high-protein, low-GI diet aiming at 25 E% of protein, 45 E% of carbohydrate, and GI < 50) and physical activity programs (high-intensity exercise 75 min/week or moderate-intensity exercise 150 min/week).

The behavior change intervention relied on a theory- and evidence-based PREVIEW Behavior Modification Intervention Toolbox (PREMIT) specifically designed for PREVIEW [25]. PREMIT offered a stage-based approach to behavior modification based on Transtheoretical Model [26]. The first 6 months included the active behavior change (learning new skills, frequent group visits), and the remaining 2.5 years was considered a behavior maintenance stage. The PREMIT behavior modification intervention was delivered in group visits organized throughout the intervention with decreasing frequency. Out of total 17 group visits, 10 were organized during the first 6 months (Supplementary Fig. 2). Even though the intervention was group-based, participants were guided within the limits of study diets and physical activity programs to make choices that best suited their personal preferences. For example, they were able to freely choose from variety of foods and exercise alternatives.

### The analytical sample of the present study

The present analysis focused on long-term maintenance of changes in eating behavior. The analytical sample included 1311 participants, who attended at least one study visit after 6 months (during the behavior maintenance stage) and provided data on at least one eating behavior (Supplementary Fig. 1).

Participants who were excluded from the analytical sample (*n* = 912, Supplementary Fig. 1) were younger and had higher BMI and perceived stress levels at baseline and at 6 months (all *p* < 0.001, Supplementary Table 1) than participants in the analytical sample. The analysis regarding weight reduction success included 962 participants who completed the study. At baseline, completers were older and had lower BMI (both *p* < 0.001) than late drop-outs (*n* = 349), who were included in the analytical sample, but did not attend the final study visit. Their perceived stress levels were also lower (*p* = 0.023).

PREVIEW intervention comprised two different study diets and eating behavior may be related to the composition of diet [27]. However, we have previously reported that there was no difference between the diet groups in changes in eating behavior dimensions [28] and to aid comprehension, we have also shown it in the present study (Supplementary figure 3). Additionally, according to accelerometer data, there was no difference in total physical activity (assessed by counts per min) between the groups [22]. Hence, participants were merged into one group irrespective of original randomization in the present analysis.

### Measurements

Only measurements that are relevant to the present analysis are described here. For further information, see the PREVIEW methodology paper [21]. Clinical investigation days were conducted throughout the intervention at the following time-points: baseline, and 2, 6, 12, 18, 24, 36 months. During these visits, anthropometry was performed and participants completed several questionnaires.

#### Eating behavior and perceived stress

Eating behavior and perceived stress were assessed using widely used and validated psychometric questionnaires: 51-item Three Factor Eating Questionnaire (TFEQ) [4] and 10-item Perceived Stress Scale (PSS) [29]. The questionnaires were self-administered and completed with computer platform during all measurement points except at 18 months.

##### Three Factor Eating Questionnaire (TFEQ)

Total scores for disinhibition (0–16 points) and hunger (0–14 points) were calculated. The original cognitive restraint scale of TFEQ was further divided to flexible and rigid dimensions (both 0–7 points) according to Westenhoefer et al. [17]. For all four scales, higher scores indicated higher tendency to the given eating behavior. Cronbach’s Alphas were calculated separately for each of the six time-points. For flexible restraint they ranged from 0.65 to 0.72, for rigid restraint from 0.43 to 0.55, for disinhibition from 0.77 to 0.82, and for hunger from 0.81 to 0.84.

##### Perceived Stress Scale (PSS)

The questionnaire contains 10 items, which are rated from 0 (never) to 4 (very often). Summary scores (range from 0 to 40 with higher scores indicating higher perceived stress) and Cronbach’s Alphas (range from 0.78 to 0.87) were calculated for each of the six time-points.

In addition to using the continuous PSS score at 6 months, we wanted to identify the participants with frequent high stress levels during the behavior maintenance stage, because it is reasonable to assume that prolonged high stress has a stronger effect on behavior. There is no established cut-off for the PSS score to screen for high stress. Hence, we identified the 20% scoring highest on the PSS at baseline, which resulted in a cut-off ≥20 for high stress. A similar relative cut-off approach has been used before [30, 31]. Frequent high stress during the intervention was defined as having high perceived stress at least two out of four measurement points between 6 months and the end of study.

#### Anthropometry and 3-year weight reduction success

Weight was measured at each time point in a fasting state, with an empty bladder, wearing underwear or other light clothing. A measurement was taken to the nearest 0.1 kg. Height was measured at the screening visit (before baseline) to the nearest 0.5 cm.

Total weight loss during the whole intervention was calculated as percentages of baseline weight ((3-year weight − baseline weight) / baseline weight × 100%). To facilitate the visualization and meaningful interpretation of the results, participants were categorized into three categories according to total weight reduction success after 3 years: (1) Successful, total weight loss >8%, (2) Partially successful, total weight loss 1–8%, and (3) Unsuccessful, total weight loss <1%.

### Statistical methods

The descriptive data were shown as mean (SD) or *n* (%) unless otherwise stated. Normality of the distributions was evaluated visually from histograms. The changes in eating behavior dimensions were analyzed using linear mixed effects models with maximum likelihood estimation. This estimation method uses all available data from all participants despite missing data at some or several time-points and it is as powerful tool to handle missing data as multiple imputation [32]. Main effects were used to analyze whether predictors (perceived stress and weight reduction success) were associated with overall levels of eating behaviors. Interaction term for predictor ∗ time was added to analyze, whether the predictor was associated with change in eating behavior. Nonsignificant interaction terms were omitted from the final reported models. To control for potential confounding, the models were adjusted for fixed effects (age at the time of signing informed consent (in years), sex, intervention diet, and eating behavior and BMI at baseline) and random effects (participant ID and intervention centers). *P*-values for fixed effects were estimated using Satterthwaite approximation for degrees of freedom [33] and *p*-values for interactions were derived from ANOVA tables.

Results of mixed models are reported as beta estimates (95% confidence interval, CI). Estimated marginal means and 95% CIs were calculated to visualize the results concerning categorical predictors. Pairwise comparisons were conducted at each relevant time point with Bonferroni adjustment. Levene’s test was used to test homogeneity of variances of eating behavior and equal variances were assumed, because the test indicated similar variances in many of the time-points. To evaluate the effect sizes, standardized beta estimates were calculated for perceived stress as continuous variable, and Cohen’s d_s_’s were calculated for group comparisons at relevant time-points [34]. Additionally, between-group Cohen’s d_s_’s were calculated for change in eating behavior in selected time periods.

Statistical analyses were conducted using the statistical program R version 4.0.3 [35] with R Studio. Package *lme4* was used to perform linear mixed effects analyses [36], and package *lmerTest* was used to obtain *p*-values for fixed effects [37]. The threshold for statistical significance was set at *p* < 0.05.

## Results

In total, 1311 participants (65% women) were included in the present analytical sample (Table 1). The mean (SD) age was 54 (10) years and baseline BMI 34.3 (5.7) kg/m^2^. During the active behavior stage (first 6 months), flexible and rigid restraint increased, and disinhibition and hunger decreased (all *p* < 0.001), but there was no change in perceived stress.

**Table 1:** Participant characteristics, eating behaviors, and perceived stress at baseline and at 6 months in PREVIEW participants who attended at least one follow-up visit after 6 month time point (*n* = 1311).

	Baseline	6 months	*p*-value (*n*)
Sex			
Women	857 (65%)	—	
Men	454 (35%)	—	
Age (years)	54 (10)	—	
BMI (kg/m^2)^	34.3 (5.7)	30.1 (5.3)	<0.001 (1290)
Missing	0	21	
Flexible restraint^a^	2.2 (1.8)	4.6 (1.8)	<0.001 (1175)
Missing	60	88	
Rigid restraint^a^	2.5 (1.6)	4.0 (1.5)	<0.001 (1190)
Missing	45	80	
Disinhibition^a^	9.1 (3.5)	7.2 (3.4)	<0.001 (1099)
Missing	104	137	
Hunger^a^	7.0 (3.6)	4.9 (3.5)	<0.001 (1097)
Missing	99	145	
Perceived stress^b^	13.1 (6.1)	13.3 (6.3)	0.219 (1158)
Missing	64	102	

Values are *n* (%) or mean (SD), and *p*-values are from paired sample *t*-test (calculated using participants, who provided both baseline and 6 months data).

^a^Measured with Three Factor Eating Questionnaire.

^b^Measured with Perceived Stress Scale.

### Perceived stress and maintenance of changes in eating behavior

Perceived stress at 6 months did not predict the changes in eating behavior from month 6 to 36, which was indicated by the nonsignificant interaction terms with time in mixed models (Table 2). However, higher perceived stress at 6 months was associated with overall lower flexible restraint and higher disinhibition and hunger from month 6 to 36 (significant main effects, all *p* < 0.001). Standardized beta estimates (95% CI) for perceived stress were −0.16 (−0.20, −0.12) on flexible restraint, 0.13 (0.10, 0.17) on disinhibition and 0.13 (0.09, 0.17) on hunger.

**Table 2:** Perceived stress at 6 months as a predictor of changes in eating behavior (EB) dimensions from month 6 to 36, results from linear mixed effects models with maximum likelihood estimation.

	Flexible restraint, *n* = 1154		Rigid restraint, *n* = 1167		Disinhibition, *n* = 1112		Hunger, *n* = 1117	
	Estimate (95% CI)	*p*-value	Estimate (95% CI)	*p*-value	Estimate (95% CI)	*p*-value	Estimate (95% CI)	*p*-value
Perceived stress	−0.051 (−0.064, −0.037)	<0.001	−0.008 (−0.019, 0.002)	0.097	0.076 (0.055, 0.098)	<0.001	0.075 (0.052, 0.098)	<0.001
Time (months)	−0.029 (−0.033, −0.026)	<0.001	−0.010 (−0.013, −0.008)	<0.001	0.023 (0.018, 0.029)	<0.001	0.022 (0.017, 0.027)	<0.001
Sex (male)	0.144 (−0.033, 0.321)	0.111	−0.140 (−0.273, −0.005)	0.041	−0.193 (−0.472, 0.087)	0.176	−0.100 (−0.390, 0.190)	0.498
Age (years)	0.003 (−0.006, 0.011)	0.517	−0.003 (−0.009, 0.003)	0.369	−0.001 (−0.014, 0.012)	0.896	0.001 (−0.012, 0.015)	0.854
Diet (MP)^a^	−0.005 (−0.169, 0.159)	0.953	0.0004 (−0.119, 0.120)	0.995	0.223 (−0.034, 0.479)	0.089	0.069 (−0.202, 0.341)	0.617
Baseline EB	0.406 (0.359, 0.454)	<0.001	0.461 (0.422, 0.500)	<0.001	0.662 (0.622, 0.702)	<0.001	0.621 (0.581, 0.660)	<0.001
Baseline BMI (kg/m^2^)	−0.051 (−0.067, −0.035)	<0.001	0.010 (−0.002, 0.021)	0.098	0.022 (−0.002, 0.047)	0.077	0.037 (0.011, 0.063)	0.005
Stress ∗ Time		0.081		0.085		0.612		0.355

The models were adjusted for random effects (participant ID and intervention center). Reported estimates for main effects are from the models without an interaction term because the interactions were not significant.

^a^Two intervention diets were high-protein, low-glycemic index and moderate-protein, moderate-glycemic index (MP).

**Fig. 1:**
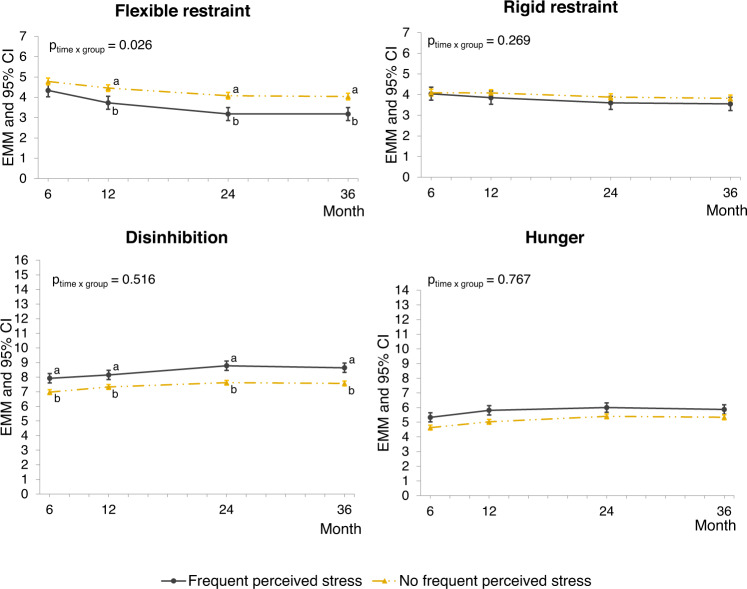
Estimated marginal means (EMM) and 95% confidence intervals (CI) of eating behaviors throughout the behavior maintenance stage of the PREVIEW intervention by stress groups. Individuals with frequent high stress levels (*n* = 132) reported high Perceived Stress Scale scores (≥20) 2 or more time-points during the behavior maintenance stage (6–36 months). Determined using linear mixed effects models with maximum likelihood estimation adjusted for age, sex, diet (high vs medium protein), and baseline (month 0) eating behavior and BMI as fixed effects and participant ID and intervention center as random effects, including group*time interaction term. Pairwise comparisons were conducted at each time point, and significantly different (*p* < 0.05, Bonferroni adjusted) mean values are indicated with different letters.

Frequent high stress was related to change in flexible restraint from month 6 to 36, as indicated by a significant group*time interaction term (*p* = 0.026). In participants with frequent stress (*n* = 132, 18%), flexible restraint decreased more compared to participants without frequent stress (*n* = 588, Fig. 1). At 6 months, there was no difference between these groups in flexible restraint, but at the end of the study, Cohen’s d_s_ (95% CI) was 0.50 (0.31–0.70). The mean (SD) change in flexible restraint from 6 to 36 months was −1.1 (2.1) in participants with frequent stress and −0.7 (1.8) in participants without frequent stress (Cohen’s d_s_ (95% CI) = 0.24 (0.04–0.43)). Regarding other eating behaviors, the changes did not differ between the groups (group ∗ time interaction terms were nonsignificant). However, frequent high stress was associated with overall higher disinhibition from month 6 to 36 (beta estimate (95% CI) = 1.00 (0.58–1.43), *p* < 0.001), and participants with frequent high stress had higher scores for disinhibition at all time-points than participants without frequent stress (Cohen’s d_s_ (95%CI) were 0.66 (0.46–0.85), 0.56 (0.36–0.75), 0.64 (0.44–0.84), and 0.67 (0.47–0.87) at months 6, 12, 24, and 36, respectively).

### Weight reduction success and eating behavior

**Fig. 2:**
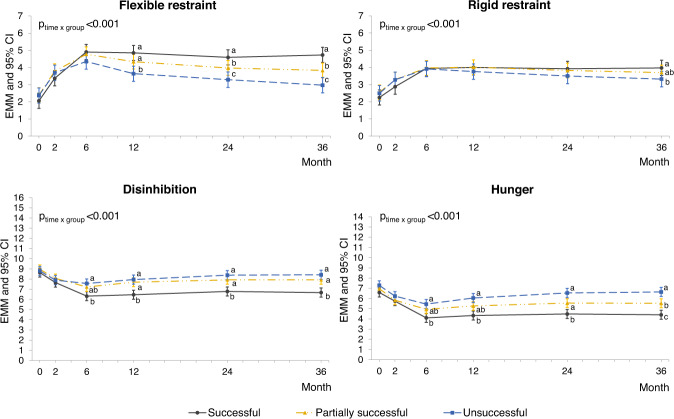
Estimated marginal means (EMM) and 95% confidence intervals (CI) of eating behaviors throughout the PREVIEW intervention by 3-year weight reduction success groups (successful weight reduction = weight loss from baseline to end of study >8% of baseline weight, *n* = 293; partially successful weight reduction = weight loss 1–8%, *n* = 405; unsuccessful weight reduction = weight loss <1%, *n* = 264). Determined using linear mixed effects models with maximum likelihood estimation adjusted for age, sex, diet (high vs medium protein), and baseline (month 0) BMI as fixed effects and participant ID and intervention center as random effects, including group*time interaction term. Pairwise comparisons were conducted at each time point, and significantly different (*p* < 0.05, Bonferroni adjusted) mean values are indicated with different letters.

Of the 962 completers, 293 (30%) were categorized as having successful, 405 (42%) partially successful, and 264 (27%) unsuccessful weight reduction at 3 years. Figure 2 illustrates that changes in all eating behavior dimensions during the 3-year intervention differed between these groups (significant group ∗ time interactions, all *p* < 0.001).

Flexible and rigid restraint increased during the first 6 months in all groups and remained higher than baseline to the end of study. However, in participants with successful weight reduction, flexible restraint remained stable after 6 months until the end of study, whereas in participants with either partially successful or unsuccessful weight reduction, flexible restraint decreased after 6 months, with a most notable decrease among participants with unsuccessful weight reduction. Regarding rigid restraint, the pattern was similar, but the effect sizes were smaller compared to flexible restraint (Supplementary table 2). Cohen’s d_s_ (95% CI) for total change from 0 to 36 months between successful and unsuccessful groups was 1.16 (0.96–1.35) for flexible restraint and 0.55 (0.37–0.72) for rigid restraint.

Disinhibition and hunger decreased from baseline to month 6 in all groups. However, the observed decreases were largest in participants with successful weight reduction. In the partially successful and unsuccessful groups, disinhibition and hunger seemed to increase slightly after 6 months, which was demonstrated with greater mean differences at the end of study compared to 6 months (Supplementary table 2). Cohen’s d_s_ (95% CI) for total change from 0 to 36 months between successful and unsuccessful groups was 0.61 (0.42–0.80) for disinhibition and 0.51 (0.32–0.70) for hunger.

## Discussion

In the present study, perceived stress after the active behavior change stage (at 6 months) did not predict subsequent change in eating behavior (i.e., maintenance of changes). However, frequent high stress during the behavior maintenance stage was associated with lapse of improved flexible restraint. Furthermore, higher perceived stress at 6 months predicted overall lower flexible restraint and higher disinhibition and hunger during the behavior maintenance stage, and frequent high stress was associated with higher disinhibition throughout the same time period.

Stress is known to be linked with obesity and eating through multiple mechanisms [11, 13, 38]. We were interested in whether high perceived stress would impair the long-term maintenance of achieved beneficial changes in eating behavior. It is reasonable to assume that under stressful situations, individual’s attention shifts towards coping with these situations, and there may not be time and energy for efforts needed to maintain achieved behavioral changes. However, stress assessed at 6 months was not associated with subsequent change in eating behavior dimensions. It must be noted that on average, participants in the present study cannot be considered particularly stressed (mean (SD) score of PSS 13.3 (6.3), range 0─40). Nonetheless, one earlier study including participants with psychological distress (mean score of 14-item PSS 25.8 (8.0)–26.9 (7.8), range 0─56) reported similar results [39]. They found that acceptance and commitment therapy intervention reduced uncontrolled eating (similar to disinhibition), but the change was not moderated by baseline perceived stress.

To capture longer term stress, we identified participants who frequently experienced high stress, and found out that they had more difficulties in maintaining increased levels of flexible restraint compared to other participants. We evaluated the effect size using Cohen’s d_s_, which indicated medium effect (0.50) between the groups at the end of study [34]. It must be noted that there are no established cut-offs for high stress or frequent high stress, which makes our definition somewhat arbitrary. Regardless, our result suggests that it may be useful to try to capture longer term high stress rather than rely on one measurement point.

Previous studies have reported cross-sectional associations between high perceived stress and less cognitive restraint [15], but higher rigid restraint [16]. Our results showed that frequent high stress was associated with a decrease in flexible restraint, as well as an overall lower flexible restraint during the behavior maintenance stage. Furthermore, perceived stress at 6 months was associated with overall lower flexible restraint. On the contrary, no association between stress and rigid restraint was found. Taken together, our results suggest that during an intervention, perceived stress may disrupt particularly flexible restraint. One possible explanation for our findings relates to flexible restraint involving gradual control of eating without for example any strictly forbidden food choices [17]. This requires continuous relative judging of food-related decisions, and hence the ability to adopt and maintain such behavior may be challenging under burdensome situations, including being under chronic stress.

The association between perceived stress and flexible restraint is interesting, because earlier research has suggested that flexible restraint may be preferable to rigid restraint in supporting long-term weight maintenance [19, 20]. In the present study, successful 3-year weight reduction was associated with both dimensions of cognitive restraint. However, the association was stronger with flexible restraint, which gives support to earlier findings [19, 20]. The benefit of cognitive restraint regarding weight management has been questioned [40, 41]. It appears that in cross-sectional studies cognitive restraint is commonly associated with higher BMI [42], but in intervention studies in participants with overweight and targeting weight loss, increases in cognitive restraint are consistently associated with beneficial outcomes [5–8, 42]. Even though our results did not imply rigid restraint being detrimental, flexible restraint appeared more important regarding weight loss maintenance. Similar observations have been made also in a very different context. A qualitative study among individuals who had maintained normal weight (BMI < 25 kg/m^2^) throughout their lives revealed that flexible eating regulation was key for their success [43]. In future interventions it may be important to pay special attention to supporting flexible ways of eating control.

We also found that perceived stress at 6 months was associated with higher disinhibition and hunger throughout the behavior maintenance stage, and frequent high stress was associated with higher disinhibition throughout the same period. Similar findings have been reported previously in cross-sectional studies [15, 16, 18]. Potential mechanisms behind these associations may include physiological responses (i.e., increased cortisol, leptin, insulin and neuropeptide Y) which promote appetite and reward value of food [13]. Individuals may also use eating as a coping mechanism (stress-induced eating) with increased preference towards palatable foods with high sugar and fat content [11, 44].

To our knowledge, this is the first study to report associations between perceived stress and repeatedly measured eating behavior over a longer time period. Many previous studies about eating behavior have included only women, but our large sample included both sexes with a broad age range (26–70 years at baseline). Moreover, the sample was multi-national and standardized measures were used across the eight study sites.

Nevertheless, the present study had also some limitations. As this is a secondary observational analysis of an intervention study, we cannot rule out the potential unmeasured confounding factors, which may cause bias. Selection bias may also be present. First, a successful ≥8% weight loss during an initial 2-month weight loss phase was required for participants to continue the study. Second, the drop-out was substantial (43% out of enrolled participants completed the study), and previous analysis showed that higher perceived stress was associated with higher likelihood of dropping out [10]. This is understandable, because taking part in an intervention is demanding, and participants who already perceive their life as stressful may be tempted discontinue the study to rule out additional responsibilities. This occurrence, however, has more likely attenuated the observed associations rather than increased them.

We applied a widely used and validated questionnaire (TFEQ) to measure eating behavior, but there are known limitations regarding the flexible and rigid restraint scales which consist of only small numbers of items. However, as expected based on the development of these two scales [17], also in the present sample rigid restraint was positively (r = 0.07, *p* = 0.010) and flexible restraint was negatively (r = −0.27, *p* < 0.001) associated with disinhibition, indicating difference between these two dimensions of cognitive restraint. Additionally, Cronbach’s Alphas for rigid restraint ranged from 0.43 to 0.55 indicating poor reliability, but it may also be related to the fact that the scale contains only seven items recorded on a 2-point scale which are factors affecting the Alpha [45]. Eating behavior and stress were self-reported predisposing to reporting bias. However, self-reporting is natural and probably the only way to capture individual’s subjective perceptions about the stressfulness of different situations in every-day life [14].

In conclusion, in the present study, stress was associated with lower levels of flexible restraint and higher tendency to overeat (disinhibition and hunger) as well as more difficulties in maintaining achieved improvement in flexible restraint. These features of eating behavior may challenge successful weight management, which was also shown in the present study. Thus, future interventions with long-term follow-ups should examine, whether incorporating stress reduction and management techniques results in a more effective treatment, at least for participants experiencing a high stress level.

## Supplementary information

Supplementary material

## Data Availability

The availability of the PREVIEW data can be queried from the leader of the project Anne Raben (ara@nexs.ku.dk).

## Code availability

The statistical code used to generate results is available upon reasonable request from the corresponding author.
