# Supplementary material for: Perceived stress as a predictor of eating behavior during the 3-year PREVIEW lifestyle intervention
Source: Nutr Diabetes. 2022 Nov 5;12:47. doi: 10.1038/s41387-022-00224-0 (PMC9637180; doi:10.1038/s41387-022-00224-0)
Supplement: Supplementary file 1 — Supplementary material [file 41387_2022_224_MOESM1_ESM.pdf]

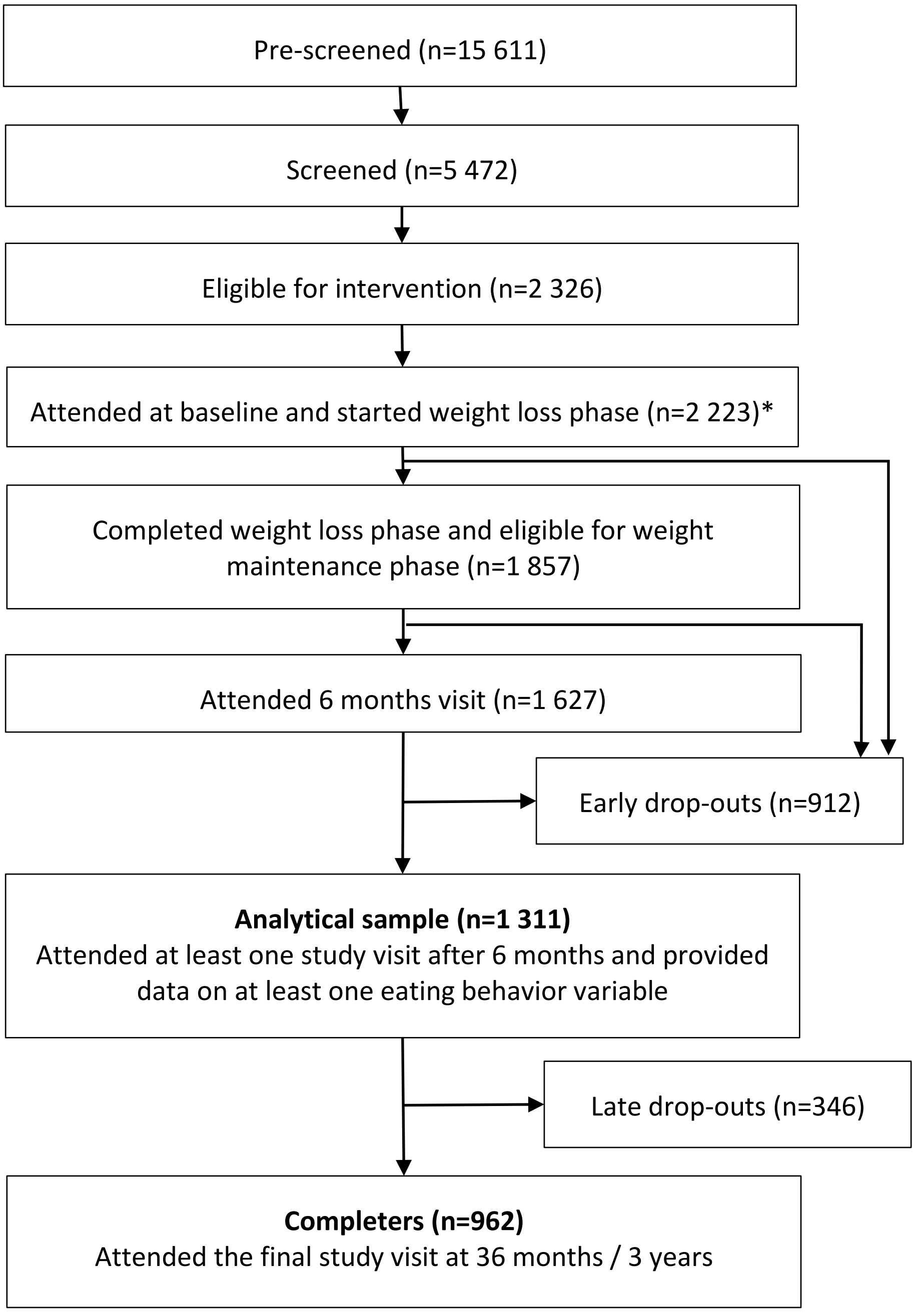

Supplementary figure 1 The PREVIEW study flow diagram (for more information, see Fogelholm *et al.* 2017 and Raben *et al.* 2021) and formulation of the present analytical sample. *A total number of participants attending the baseline visit was 2 224, but one individual withdrew a consent during the intervention and requested all data to be deleted.

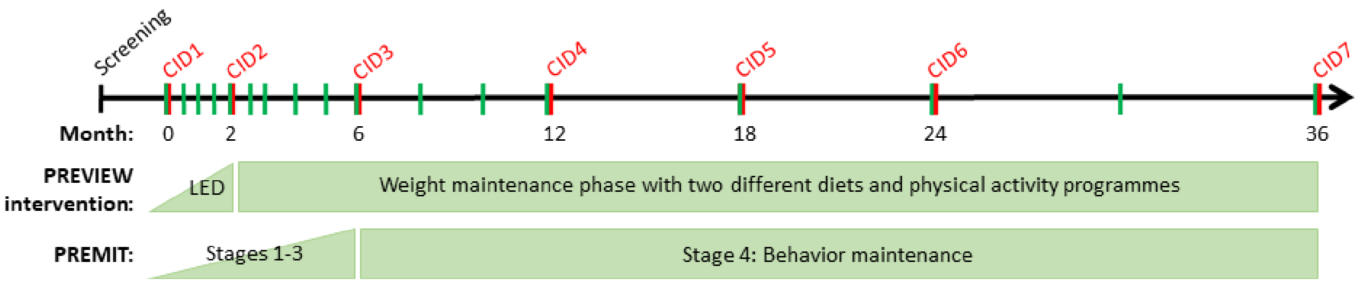

Supplementary figure 2 Overview of the PREVIEW intervention with 17 group meetings (marked in green) and seven clinical investigation days (CIDs, marked in red). The behavior change intervention was administered according to PREVIEW Behavior Modification Intervention Toolbox (PREMIT) designed for PREVIEW (Kahlert *et al*. 2016). Active behavior change was the goal during the first six months which were divided for preliminary, preparation and action stages (Stages 1-3) and behavior maintenance was the goal during the rest of the intervention considered as the behavior maintenance stage. LED = low energy diet.

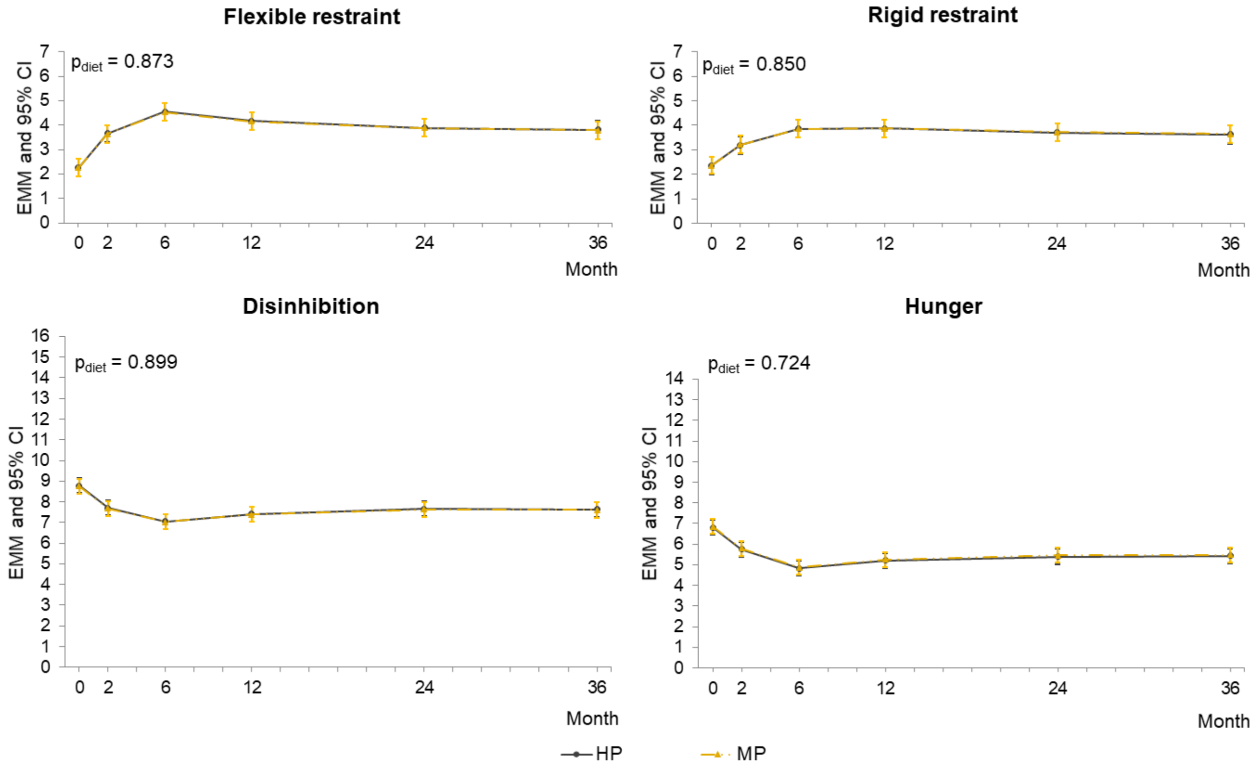

Supplementary figure 3 Estimated marginal means (EMM) and 95% confidence intervals (CI) of eating behaviors throughout the PREVIEW intervention by diet groups (HP= high protein, low glycemic index, n=674 at baseline; MP = medium protein, medium glycemic index, n=637 at baseline). Determined using linear mixed effects models with maximum likelihood estimation adjusted for age, sex, and baseline (month 0) BMI as fixed effects and participant-ID and intervention centre as random effects. Diet x time interaction terms were not significant and hence not included in the models.

Supplementary table 1 Participant characteristics, eating behaviors and perceived stress at baseline and at 6 months. Participants categorized according to time spent in the PREVIEW study. Analytical sample includes late drop-outs and completers. P-values from t-test for continuous variables and chi^2-test for sex.

	Early drop-outs[a], n=912	Late drop-outs[b], n=349	Completers, n=962	Early drop-outs vs. Analytical sample[c] p-value	Late drop-outs vs. Completers p-value
Sex				0.007	0.195
Women	646 (71%)	238 (68%)	619 (64%)		
Men	266 (29%)	111 (32%)	343 (36%)		
Age	48 (12)	52 (11)	55 (10)	<0.001	<0.001
**Baseline**					
BMI	36.8 (7.4)	36.6 (6.2)	33.5 (5.3)	<0.001	<0.001
Flexible restraint	2.2 (1.9)	2.1 (1.9)	2.3 (1.8)	0.883	0.059
Missing	61	18	42		
Rigid restraint	2.4 (1.6)	2.5 (1.6)	2.6 (1.6)	0.018	0.334
Missing	58	16	29		
Disinhibition	9.2 (3.6)	9.3 (3.5)	9.0 (3.5)	0.615	0.186
Missing	88	35	69		
Hunger	7.1 (3.6)	6.9 (3.7)	7.0 (3.5)	0.450	0.643
Missing	88	31	68		
Perceived stress	15.4 (6.5)	13.7 (6.0)	12.9 (6.1)	<0.001	0.023
Missing	45	13	51		
**Month 6**					
BMI	33.1 (6.0)	32.2 (5.7)	29.4 (4.9)	<0.001	<0.001
Missing	631	16	5		
Flexible restraint	4.0 (2.0)	4.4 (1.9)	4.7 (1.8)	<0.001	0.008
Missing	657	45	43		
Rigid restraint	3.8 (1.6)	3.9 (1.5)	4.1 (1.5)	0.010	0.200
Missing	654	38	42		
Disinhibition	7.9 (3.9)	7.3 (3.6)	7.2 (3.3)	0.003	0.491
Missing	677	61	76		
Hunger	5.4 (3.6)	5.0 (3.5)	4.9 (3.5)	0.047	0.675
Missing	678	57	88		
Perceived stress	15.0 (6.5)	14.2 (6.5)	13.0 (6.2)	<0.001	0.003
Missing	662	46	56		

a) Participant discontinued the study before one year study visit
b) Participant discontinued the study after one year study, and did not attend the final 3 year study visit
c) Analytical sample includes late drop-outs and completers (n=1 311)

Supplementary table 2 Mean (SD) eating behavior scores at selected time points and total change during the whole intervention (from month 0 to 36) according to 3-year weight reduction success.

	Successful n=293	Partially successful n=405	Unsuccessful n=264	Successful vs. Partially successful) Cohen's d_s (95% CI)	Successful vs. Unsuccessful Cohen's d_s (95% CI)
**Flexible restraint**					
At 36 months	4.7 (1.8)	3.9 (2.1)	2.9 (2.0)	0.40 (0.25–0.56)	0.93 (0.74–1.11)
Total change	2.7 (1.8)	1.4 (1.9)	0.6 (1.9)	0.67 (0.51–0.84)	1.16 (0.96–1.35)
**Rigid restraint**					
At 36 months	4.0 (1.5)	3.8 (1.6)	3.5 (1.5)	0.13 (-0.03–0.28)	0.36 (0.19–0.54)
Total change	1.7 (1.6)	1.2 (1.4)	0.9 (1.6)	0.38 (0.22–0.54)	0.55 (0.37–0.72)
**Disinhibition**					
At 6 months	6.3 (3.1)	7.3 (3.3)	7.9 (3.4)	0.30 (0.15–0.46)	0.50 (0.33–0.68)
At 36 months	6.6 (3.3)	8.0 (3.7)	8.9 (3.9)	0.38 (0.22–0.54)	0.63 (0.45–0.81)
Total change	-2.0 (2.9)	-1.0 (2.9)	-0.3 (2.6)	0.33 (0.17–0.50)	0.61 (0.42–0.80)
**Hunger**					
At 6 months	4.1 (3.3)	5.0 (3.4)	5.7 (3.6)	0.25 (0.09–0.41)	0.46 (0.28–0.64)
At 36 months	4.3 (3.4)	5.5 (3.7)	6.8 (3.9)	0.32 (0.16–0.48)	0.69 (0.51–0.88)
Total change	-2.2 (3.1)	-1.4 (2.7)	-0.6 (3.0)	0.25 (0.08–0.41)	0.51 (0.32–0.70)

Successful = weight loss from baseline to the end of study >8% of baseline weight; partially successful = weight loss 1-8%; unsuccessful = weight loss < 1%
